# Mitochondria in depression: The dysfunction of mitochondrial energy metabolism and quality control systems

**DOI:** 10.1111/cns.14576

**Published:** 2024-02-09

**Authors:** Mengruo Jiang, Liyuan Wang, Hui Sheng

**Affiliations:** ^1^ College of Basic Medicine Naval Medical University Shanghai China; ^2^ Department of Physiology, College of Basic Medicine Naval Medical University Shanghai China

**Keywords:** depression, energy metabolism, mitochondria, mitochondrial quality control system

## Abstract

**Background:**

Depression is the most disabling neuropsychiatric disorder, causing difficulties in daily life activities and social interactions. The exact mechanisms of depression remain largely unclear. However, some studies have shown that mitochondrial dysfunction would play a crucial role in the occurrence and development of depression.

**Aims:**

To summarize the known knowledge about the role of mitochondrial dysfunction in the pathogenesis of depression.

**Methods:**

We review the recent literature， including 105 articles, to summarize the mitochondrial energy metabolism and quality control systems in the occurrence and development of depression. Some antidepressants which may exert their effects by improving mitochondrial function are also discussed.

**Results:**

Impaired brain energy metabolism and (or) damaged mitochondrial quality control systems have been reported not only in depression patients but in animal models of depression. Although the classical antidepressants have not been specially designed to target mitochondria, the evidence suggests that many antidepressants may exert their effects by improving mitochondrial function.

**Conclusions:**

This brief review focuses on the findings that implicate mitochondrial dysfunction and the quality control systems as important etiological factors in the context of depressive disorders. It will help us to understand the various concepts of mitochondrial dysfunction in the pathogenesis of depression, and to explore novel and more targeted therapeutic approaches for depression.

## Graphical abstract

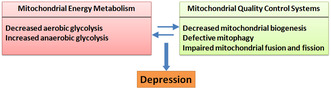

## 1 INTRODUCTION

Depression is a prevalent psychiatric disorder characterized by loss of interest, negative rumination, and fatigue, with high morbidity, mortality, and disability.^1^ It has become a major public health problem, bringing tremendous burdens to the world.^2^ It is accepted that multiple mechanisms may contribute to depression, including monoamine neurotransmitter deficiency, Hypothalamic–pituitary–adrenal (HPA) axis dysregulation, microbiome–brain–gut axis abnormality, and immuno‐inflammatory overactivation.^3^ Despite there being a variety of available antidepressants, more than one‐third of patients are refractory to these drugs.^4^, ^5^ Thus, further exploration is needed to uncover the pathophysiological mechanisms underlying depression.

Mitochondria are ubiquitous double membrane‐bound organelles found in almost all eukaryotic cells. They are most prominently known for playing a pivotal role in energy production and being involved in complex physiological activities such as energy metabolism, cell survival, and nervous system development.^6^ Moreover, mitochondrial dysfunction not only impairs energy production but can also be linked with metabolic and neuropsychiatric disorders such as depression.^4^ The concept that mitochondrial dysfunction is one of the causes of depression, is supported by a wide range of studies on cell cultures, animal models, and clinical researches.^5^, ^7^, ^8^, ^9^ The review will focus on the findings that implicate abnormalities in mitochondrial morphology and function, as well as the resultant quality control systems as important etiological factors in the context of depressive disorders.

## 2 MITOCHONDRIAL MORPHOLOGY AND DEPRESSION

Some studies have demonstrated that abnormal mitochondrial structure may disrupt its function, and then contribute to various brain disorders, including depression.^10^, ^11^, ^12^ Previous studies from bipolar disorder patients have shown that neurons in the postmortem prefrontal cortices have significantly a larger number of small mitochondria, whereas mitochondria in peripheral cells display an abnormal pattern of clumping and marginalization in the intracellular distribution, suggesting subtle changes in the critical network architecture of mitochondria in the neurons.^10^, ^13^ The similar results have been revealed in the animal models. Gebara et al.^12^ demonstrated that high‐anxious rats exhibit more severe depression‐like behavior, and present larger mitochondria area and mitochondria tissue coverage as well as a higher number of mitochondria‐mitochondria contacts in the medium spiny neurons from the nucleus accumbens (NAc). As we all know, chronic unpredictable mild stress (CUMS) is a well‐established model of depression, which can induce long‐term behavioral disturbances that resemble symptoms of clinical depression.^14^, ^15^, ^16^ Some studies revealed that CUMS can result in abnormal mitochondrial morphology, such as the mitochondrial rupture and impairment in hippocampal astrocytes, and the significant mitochondrial swelling, broken mitochondrial cristae, and decreased mitochondrial matrix density in hippocampus.^17^, ^18^ Additionally, our previous study demonstrated that the prenatal dexamethasone (Dex) exposure leads to depression‐like behavior and mitochondrial damage in hippocampus, including a decrease in cristae density or even the disappearance, vacuole formation by the mitochondrial outer membrane extension, as well as the intermembrane space expansion, which can be improved by exercise.^11^ Taken together, these studies corroborate the close coupling between the abnormal mitochondrial morphology and the development of depression.

## 3 MITOCHONDRIAL ENERGY METABOLISM AND DEPRESSION

As mentioned, mitochondria are bioenergetic organelles that meet most of the energy needs of organisms by providing adenosine triphosphate (ATP). The metabolism of glucose, lipids, and proteins is connected through the tricarboxylic acid (TCA) cycle and oxidative phosphorylation, which also requires mitochondria.^19^ Of note, mitochondria are particularly important for brain because of both its high levels of energy use and inability to store large amounts of energy reserves in the form of glycogen. As glucose metabolism supplies more than 95% of ATP in the brain, this may strongly suggests the intriguing possibility that decreased ATP level caused by mitochondrial disorders is critically involved in neurological diseases, including depression.^20^, ^21^

In the past decade, evidence from some patients with major depressive disorder (MDD) exhibits decreased glucose metabolism level in some brain regions related to emotion regulation, such as bilateral insula, left lentiform nucleus putamen and extra‐nuclear, right caudate and cingulate gyrus.^22^ Similarly, several studies have shown that the beta‐nucleoside triphosphate, which arises from beta‐ATP, is much lower in the basal ganglia of MDD patients.^23^, ^24^ Notably, it has also been found that patients with diabetes have a higher risk of depression, and the co‐occurrence of diabetes mellitus and depression may be associated with abnormal glucose metabolism and the subsequent decreased ATP in the brain.^25^, ^26^ In accordance with human researches, many studies from animal models of depression have also shown that there may be a link between mitochondrial dysfunction and depression.^27^, ^28^, ^29^ For instance, it was reported that CUMS not only induces depression but reduces the ATP content of the prefrontal cortex and hippocampus in mice.^30^, ^31^ Cao et al.^32^ found that chronic social defeat stress reduces ATP abundance in some brain regions, while the lateral intracerebroventricular injection of ATP, or an increase in endogenous ATP released by astrocytes, can significantly improve the depression‐like behavior in the mice. Moreover, the study by Alhassen et al.^33^ demonstrated that acetyl‐L‐carnitine supplementation can improve mitochondrial function and promote ATP production, thereby ameliorating depression caused by intergenerational trauma. In addition, it has been shown that prenatal Dex exposure results in depression‐like behavior, accompanied by mitochondrial dysfunction and the diminished production of ATP in the frontal cortices or hippocampus of the adult rodents, and that improvement of mitochondrial function in brain improves the depression‐like behavior.^11^, ^21^, ^34^

**FIGURE 1:**
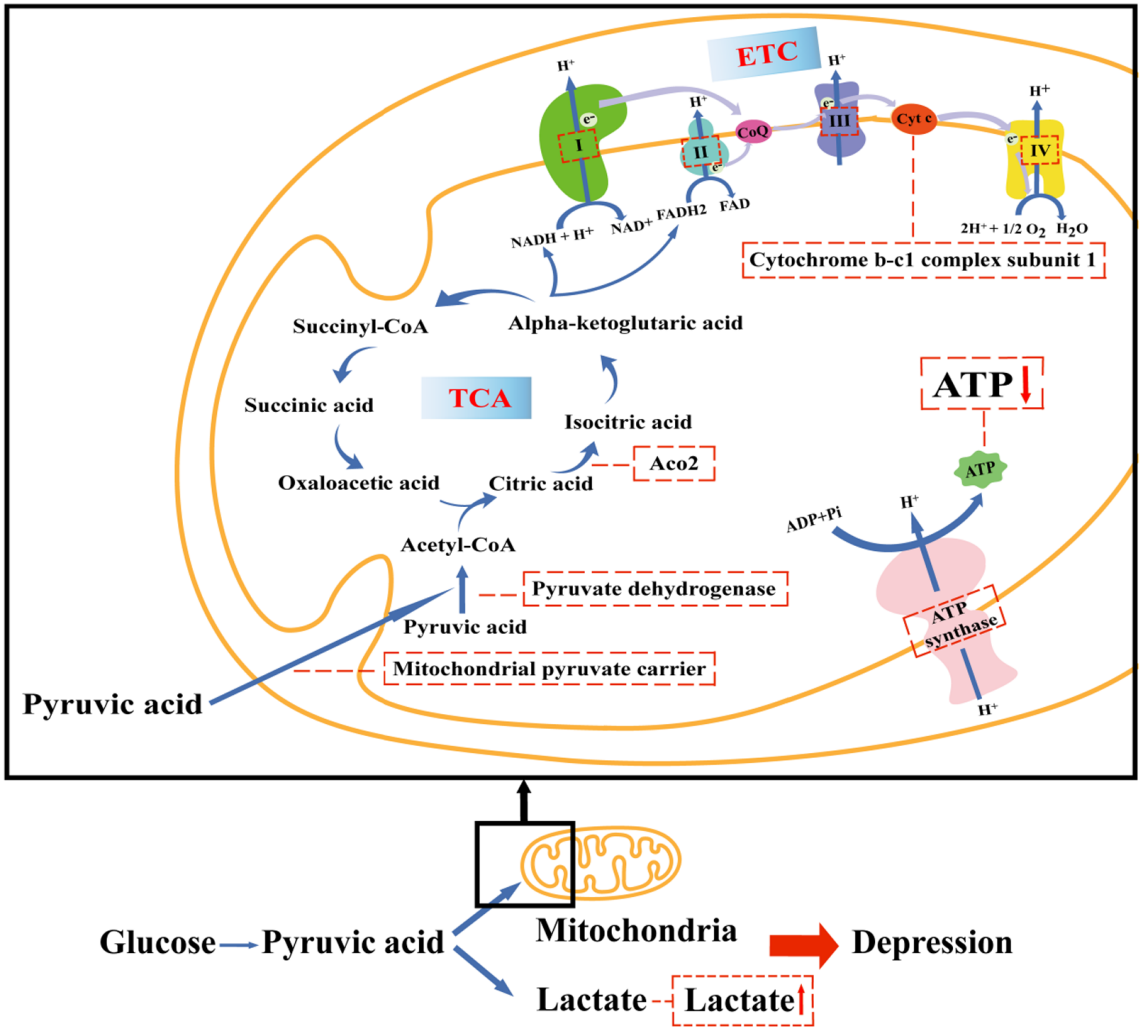
Schematic diagram of mitochondrial energy metabolism and depression. The blue arrows represent the processes of aerobic and anaerobic glycolysis, and the purple arrows represent electron transport in the electron transport chain (ETC) reaction. The ETC is localized within the inner mitochondrial membrane or cristae of the mitochondria and is composed of five multimeric protein complexes (mitochondrial respiratory chain complex I–IV and ATP synthase) responsible for ATP production by oxidative phosphorylation. The red dashed boxes represent the dysfunction of energy metabolism, which is proved to lead to depression. ADP, adenosine diphosphate; ATP, adenosine triphosphate; CoQ, coenzyme Q; Cyt c, cytochrome c; e‐, electron; FAD, flavin adenine dinucleotide; I–IV, mitochondrial respiratory chain complex I–IV; NAD, nicotinamide adenine dinucleotide; TCA, tricarboxylic acid cycle.

Some studies from human and animals suggest that the deficiency of ATP resulting from mitochondrial disorders contributes to the pathogenesis of depression. It is widely known that ATP is mainly produced by aerobic and anaerobic glycolysis. The former is a series of reactions wherein glucose is converted to pyruvate through glycolysis in the cytoplasm, and pyruvate is subsequently oxidized in the mitochondria to generate ATP through the TCA cycle and oxidative phosphorylation, while the later includes glycolysis and lactic acid production, which mainly takes place in the cytoplasm.^6^ Due to the high energy demands of the brain, the processes of aerobic and anaerobic glycolysis are critical for the proper functioning of the brain, including emotion. Then the roles of aerobic and anaerobic glycolysis in the development of depression are discussed in the following section (Figure 1).

### 3.1 Aerobic glycolysis

Mitochondrial oxidative phosphorylation is one of the important processes in aerobic glycolysis and requires the mitochondrial respiratory chain, which is composed of various respiratory enzyme complexes and located in a special structure of the inner mitochondrial membrane.^35^ Abnormality of the mitochondrial respiratory chain has been considered to play an important role in the development of neuropsychiatric diseases, such as depression.^36^, ^37^, ^38^ For instance, postmortem studies showed that the levels of mitochondrial respiratory chain complex I subunits NDUFV1, NDUFV2, and NADUFS1 are reduced in the lateral cerebellar hemisphere of patients with MDD.^39^, ^40^ Moreover, both mitochondrial respiratory chain complex I and III are also observed to be significantly decreased in the neuron‐derived extracellular vesicles (NDEVs) of MDD patients.^41^ Similarly, some animal studies verified that the activity of mitochondrial respiratory chain complex II and V, and the levels of some mitochondrial‐respiration proteins, such as cytochrome b‐c1 complex subunit 1, ATP synthase subunit O and ATP synthase γ chain, are significantly inhibited in the prefrontal cortex of depressive rodents.^30^, ^42^ Of note, Damri et al.^38^ reported that intraperitoneal administration of rotenone, an inhibitor of mitochondrial respiratory chain complexes, causes depression‐like behaviors in mice, which is accompanied by abnormal changes in mitochondrial‐respiration proteins levels in hippocampus.

Recently, it has been shown that TCA cycle in aerobic glycolysis is also involved in the development of depression. Linghu et al.^43^ revealed depressive rats caused by CUMS show significantly decreased TCA cycle and increased gluconeogenesis pathway, which may be related to the decrease of mitochondrial pyruvate carrier. It has also been shown that the level of pyruvate dehydrogenase, which is a key enzyme linking glycolysis with the TCA cycle, is significantly decreased in the frontal cortex of prenatal Dex‐induced depressive rats. Furthermore, it has been revealed the downregulation of Aco2 in chronic social isolation‐sensitive rats, which would imply that the TCA cycle is abnormal.^44^ Although these findings provide evidence that inhibition of TCA cycle is present in some animal models of depression, it is very hard to explain their connections at current stage.

### 3.2 Anaerobic glycolysis

Anaerobic glycolysis often occurs in occasions when the regeneration of ATP in mitochondria is insufficient to meet the requirement, such as ischemia, hypoxia, or mitochondrial dysfunction.^45^ Indeed, in mitochondrial dysfunction conditions, the process of glucose catabolism is impaired, which affects the Krebs cycle and leads to the increase of anaerobic glycolysis, eventually resulting in lactate accumulation.^46^ Therefore, lactate accumulation is a key indicator of increasing anaerobic glycolysis.

In fact, elevated lactate concentration is common in patient with depression.^47^, ^48^, ^49^ For example, some MDD patients exhibit higher concentration of lactate in the pregenual anterior ingulated cortex, which is proved to be positively correlated with depression severity.^47^, ^49^ Moreover, some patients with MDD or bipolar disorder also have increased lactate concentration in ventricular cerebrospinal fluid (CSF).^48^ Notably, the measurement of lactate concentration in CSF has been considered to be helpful to identify some types of depression. It has been believed that the accumulation of lactate is associated with increased fraction of energy utilization via glycolysis and reduced mitochondrial oxidative clearance of lactate in MDD patients.^47^ Consistent with human studies, depression model rodents also showed the similar results. For instance, study from Liu et al.^50^ demonstrated that CUMS increases lactate concentration in hippocampus in rats. Detka et al.^51^ reported that the lactate concentration in the frontal cortex is significantly higher in animals subjected to prenatal stress, which indicates that prenatal stress enhances the synthesis of lactate and glycolysis in response to adverse stimuli acting later in life. The enhanced glycolysis may be a mechanism to compensate for the weakness of the Krebs cycle or oxidative phosphorylation. The data supports the conclusions that lactate accumulation is in conjunction with depression, which suggests the abnormalities of anaerobic glycolysis are involved in depression. Nevertheless, it remains to be investigated in future studies.

## 4 MITOCHONDRIAL QUALITY CONTROL SYSTEMS AND DEPRESSION

Mitochondria are not static entities; they need quality control systems to maintain their structural and functional integrity. Mitochondrial quality control systems include mitochondrial biogenesis, mitophagy, fusion and fission, etc., which are essential for the calcium homeostasis, neuroplasticity, cell survival, and nervous system development.^52^ A growing body of studies suggests that disturbances of mitochondrial quality control systems, such as decreased mitochondrial biogenesis, defective mitophagy, as well as impaired mitochondrial fusion and fission, are all involved in the development and progress of depression.^53^, ^54^, ^55^ (Table 1).

**TABLE 1:** The changes of proteins related to mitochondrial quality control systems in studies of depression.

Mitochondrial quality control systems	Proteins	Sampling material	Upregulated/downregulated	References
Mitochondrial biogenesis	PGC‐1α	Human	↓	56, 57
		CMS mice	↓	55
		CUMS rats	↓	53, 58
		High‐fat diet mice	↓	59
	NRF 1	Human	↓	57
	NRF 2	Human	↓	41
	AMPK	Dex‐induced rats	↓	60
	SIRT1	Conditional SIRT1 knockout mice	SIRT1 knockout	61
	TFAM	Human	↓	57
Mitophagy	PINK1	Human (Parkinson's disease patients)	Parkin mutation	62
		PINK1−/−mice	PINK1 knockout	63
	Parkin	Human	↓	54, 64
		Primary hippocampal astrocytes of CMS mice	Parkin translocation↓	17
	BNIP3L/NIX	Glucocorticoid‐induced hippocampal neurons, SH‐SY5Y cells, and Mice	↓	65
		CUMS mice	↓	66
Mitochondrial fusion	Mfn1	Prenatally stressed rats	↑	67
		CUMS/Glucocorticoids induced rats	↓	68
		Streptozotocin‐induced diabetic mice	↓	69
	Mfn2	Prenatally stressed rats	↑	67
		PBMCs of MDD patients	↑	54
		NDEVs of MDD patients	↓	41
		Outbred rats	↓	12
		Streptozotocin‐induced diabetic mice	↓	69
	Opa1	PBMCs of MDD patient	↑	54
		Streptozotocin‐induced diabetic mice	↓	69
Mitochondrial fission	Drp1	CUMS mice	↑	70
		Maternal separation rats	↑	71
	Fis1	Maternal separation rats	↑	71

Abbreviations: AMPK, 5′‐AMP activated protein kinase; BNIP3L/NIX, BCL2 interacting protein 3 like; CMS, chronic mild stress; CUMS, chronic unpredictable mild stress; Drp, Dynamin‐related protein; Fis, Fission protein; MDD, major depressive disorder; Mfn, Mitofusin; NDEVs, neuron‐derived extracellular vesicles; NRF, nuclear respiratory factor; Opa, Optic Atrophy; PBMCs, peripheral blood mononuclear cells; PGC‐1α, peroxisome proliferator‐activated receptor‐γ coactivator 1α; PINK, PTEN‐induced putative kinase; SIRT, Sirtuin; TFAM, mitochondrial transcription factor A.

### 4.1 Mitochondrial biogenesis

Mitochondrial biogenesis is a process generating new mitochondrial offspring to maintain an adequate mitochondrial number, therefore to compensate the ATP deficiency or energy crises.^72^ Disrupted mitochondrial biogenesis diminishes the mitochondrial mass, accelerates mitochondrial senescence, and then promotes mitochondrial dysfunction. An increasing body of evidence indicates that the main factors affecting mitochondrial biogenesis include transcriptional coactivator peroxisome proliferator‐activated receptor‐γ coactivator 1α (PGC‐1α), nuclear respiratory factor 1 (NRF1), nuclear respiratory factor 2 (NRF2), 5′‐AMP activated protein kinase (AMPK), Sirtuin1 (SIRT1), mitochondrial transcription factor A (TFAM), etc.^56^, ^57^ The alterations of these above‐mentioned factors may interrupt mitochondrial biogenesis, and then induce mitochondrial dysfunction, eventually leading to depression.^41^, ^53^, ^57^, ^58^, ^73^

It is known that PGC‐1α, termed the “master regulator of mitochondrial biogenesis,” plays an important role in the etiology of depressive disorders. For instance, it was reported that some patients with psychotic unipolar depression exhibit lower PGC‐1α level in whole‐blood.^56^ Similarly, the expression of PGC‐1α and its downstream genes TFAM and NRF1, is observed to be downregulated in blood monocyte cells in patients with MDD.^57^ Interestingly, an animal study performed by Yan et al.^55^ demonstrated that PGC‐1α has been implicated in stress and resilience to stress‐induced depression‐like behaviors. Yang et al.^59^ reported that a high‐fat diet can induce depression‐like behavior in mice through leading to inhibition of the CREB/PGC‐1α signal pathway and mitochondrial dysfunction in hippocampus. Additionally, the study from Hu et al.^58^ has shown that CUMS rats show the disrupted mitochondrial biogenesis in hippocampus, which is closely linked to the reduction of PGC‐1α, ERRα, and FNDC5, and the BDNF expression and inhibition of PGC‐1α/FNDC5/BDNF pathway. Similarly, Wu et al.^53^ have reported that the expression of PGC‐1α is decreased in the hippocampus of CUMS rats, and curcumin, a traditional Chinese medicine ingredient, is able to alleviate the depression‐like behavior by enhancing the expression of PGC‐1α and promoting mitochondrial biogenesis. The study by Hu et al.^74^ also demonstrated that the novel antidepressant Esketamine could provide an noticeable antidepressant effect through activating PGC‐1α/irisin/ERK1/2 signaling pathway in the hippocampus of CUMS mice. Taken together, PGC‐1α and its signaling pathway play an important role in depression, which may be related to mitochondrial biogenesis.

Consistent with PGC‐1α, other genes, such as NRF2, AMPK and SIRT1 are also associated with mitochondrial biogenesis and depression.^41^, ^61^ It is reported that NRF2 might promote the expression of TFAM and drive transcription and replication of mtDNA, and some patients with MDD frequently show lower NRF2 level in NDEVs.^41^ Moreover, it has been shown that the pAMPK level, which acts as an energy sensor of the cell and a key regulator of mitochondrial biogenesis, is significantly inhibited in hippocampus and prefrontal cortex of depressive rats caused by Dex exposure.^60^ In addition, Lei et al.^61^ found that the ablation of SIRT1 in cortical and hippocampal glutamatergic neurons can reduce mitochondrial density and mitochondrial biogenesis, resulting in depression‐like behavior in male mice, while SIRT1 activator promotes mitochondrial biogenesis and exhibits an antidepressant‐like effect. Conversely, Kim et al.^75^ found that chronic social defeat stress increases SIRT1 levels in the NAc, which mediates depression‐ and anxiety‐like behaviors. These conflicting results may be due to differences in specific brain regions, cell type‐specific roles, signaling pathways downstream, and rodent model used. Further work is needed to address this complexity.

### 4.2 Mitophagy

Mitophagy is a vital pathway that selectively removes defective mitochondria through the process of autophagy, which can affect mitochondrial quality and quantity, thereby maintaining mitochondrial and cellular homeostasis.^76^ Indeed, an impairment of the mitophagy pathway may trigger the gradual accumulation of defective mitochondria, then lead to mitochondrial dysfunction, and further participate in the development of neuropsychiatric diseases such as depression.^77^ Data from some MDD participants shows that the clearance of damaged mitochondria is impaired in peripheral blood mononuclear cells (PBMCs) through assessing some markers of mitochondria.^54^ In animal models of depression, it is reported that although many autophagosomes are found in the hippocampal neurons of CUMS mice, but neither mitophagosomes nor mitolysosomes are seen, which indicates that the mitophagy is inhibited by CUMS.^66^ Similarly, Wang et al.^78^ demonstrated that CUMS severely disrupts mitophagy in hippocampus, while elevated mitophagy can ameliorate depression‐like behavior in mice.

It is generally accepted that mitophagy pathways include the PTEN‐induced putative kinase 1 (PINK1)/Parkin pathway and the receptor‐mediated mitophagy pathway. The PINK1/Parkin pathway refers to PINK1 recruits Parkin to the depolarized mitochondria, and Parkin promotes mitophagy via the ubiquitination of outer mitochondrial membrane proteins and the recruitment of Ub‐binding autophagic components, which is believed to be the ubiquitin (Ub)‐dependent pathway.^79^ Some studies have reported that low PINK1/Parkin levels and the subsequent mitophagy inhibition may be important pathogenic factors for depression.^17^, ^63^ Recent studies have shown that some MDD patients have lower level of Parkin in the PBMCs.^54^, ^64^ Interestingly, from the perspective of gene mutation, higher depression index has been reported in Parkinson's disease patients with homozygous or compound heterozygous Parkin mutations, suggesting there may be a close relationship between this mitophagy pathway and depression.^62^ In animal models, it is demonstrated that PINK1 deficiency decreases the threshold for chronic stress‐induced depression, indicating that defective mitophagy is linked to the pathogenesis of depression.^63^ TOMM20 is found to be a mitochondrial outermembrane protein which can be degraded by E3 ubiquitin ligase Parkin recruited by PINK1. Shu et al.^17^ reported that glucocorticoid inhibits the mitochondrial translocation of Parkin and TOMM20 degradation in primary hippocampal astrocytes of chronic mild stress mice model, which disrupts the process of mitophagy and contributes to depression.

More recently, several mitophagy receptors (Ub‐independent pathway) can directly induce mitophagy, which include outer mitochondrial membrane (OMM) proteins such as BCL2 interacting protein 3 like (BNIP3L/NIX or NIX), BCL2 and adenovirus E1B 19‐kDa‐interacting protein 3 (BNIP3).^80^ A growing body of studies has shown that these OMM proteins participate in the process of autophagy and mitophagy, and have a wide range of physiological and behavioral effects.^65^, ^81^ However, few researches focus on the effects of OMM proteins on depression. For example, Choi et al.^65^ revealed that glucocorticoids exposure, which is the well‐known risk factor of depression, damages NIX‐dependent mitophagy and subsequent synaptic homeostasis, while the restoration of NIX level reverses this effect. More recently, Jin et al.^66^ reported that CUMS inhibits the NIX expression and mitophagy in hippocampus, eventually leading to the depression‐like behavior in mice. And they also found that the enhancement of NIX‐mediated mitophagy ameliorates the depression induced by CUMS.

### 4.3 Mitochondrial fusion and fission

Mitochondrial fusion is a process that allows content mixing between intact and dysfunctional mitochondria, whereas fission is the process that includes sequestration of irreversibly damaged, fusion‐incompetent mitochondria and their subsequent elimination by autophagy.^82^ As replacement of damaged mitochondria contributes to the integrity and homogeneity of the mitochondrial population in a cell, both mitochondrial fusion and fission are coordinated to maintain homeostasis of the mitochondria in the cellular stress response. Therefore, erroneous mitochondrial fission or fusion promotes the formation of mitochondrial fragments that contain damaged mitochondrial DNA, and exhibits impaired oxidative phosphorylation.^83^ It has been illustrated that the levels of mitofusin (Mfn) 1 and 2, and Optic Atrophy 1 (Opa1) are involved in mitochondrial fusion, whereas the level of dynamin‐related protein 1 (Drp1) and fission protein 1 (Fis1) are involved in mitochondrial fission. The aberrant expression of these genes reflects impaired mitochondrial fusion and fission.^84^

Mitochondrial fusion mainly depends on distinct mitochondrial sublocalization of three fusogenic proteins: the OMM‐located Mfn1 and Mfn2, and the inner mitochondrial membranes (IMM)‐located Opa1. Mfn1 and Mfn2 are transmembrane GTPases located on the mitochondrial outer membrane, acting as key mediators of mitochondrial fusion.^85^ Clinical research has shown that Mfn2 level is reduced strikingly in the NDEVs of MDD patients, but it can be reversed through treatment of a selective serotonin reuptake inhibitor (SSRI).^41^ However, Scaini et al.^54^ reported that some MDD patients have increased levels of Mfn2 and Opa1 in the PBMCs. It suggests that mitochondrial fusion‐related proteins expression is complicated in depressive patients. Interestingly, in rodents, it was reported that Mfn2 expression is downregulated in the NAc of outbred rats, which show depression‐like and anxiety‐like behaviors.^12^ According to the studies of Liu et al.,^68^ CUMS or glucocorticoids treatment decreases the expression of Mfn1 and Mfn2 in the cerebral cortex of rats, which regulates depression‐like behavior. Additionally, it is also demonstrated that the expression of mitochondrial fusion genes Mfn1, Mfn2, and Opa1 is decreased in hippocampus and frontal cortex of depressive mice comorbid with diabetes.^69^ Nevertheless, Feng et al.^67^ found that the expression of Mfn1 and Mfn2 is increased in the hippocampus of depressive rats caused by prenatal restraint stress, indicating prenatal stress promotes mitochondrial fusion. The differences in experimental subjects and conditions may be responsible for this discrepancy, in particular, the heterogeneous expression of mitochondrial fusion proteins in different types of depression or animal models of depression.^54^

Prior studies have demonstrated that some factors, such as Drp1and Fis1, are the key mediators to trigger mitochondrial fission. Drp1, a GTPase, can be recruited to the mitochondrial outer membrane to regulate mitochondrial fission in cellular stress response.^86^ Fis1, a mitochondrial outer membrane protein, participates in the recruitment of Drp1 through its cytosolic domain, and plays a similar role in mitochondrial fission as Drp1.There is few studies on the relationship between mitochondrial fission and depression. Recently, Tabassum et al.^70^ have demonstrated Drp1 level is significantly increased in hippocampus of depressive mice induced by CUMS. Moreover, Deng et al.^71^ have also shown that both Drp1 and Fis1 levels are increased in the hippocampus of depressive rats induced by maternal separation, while the antidepressant administration can reverse the abnormal expression of Drp1 and Fis1.

## 5 ANTIDEPRESSANTS AND MITOCHONDRIA

Most classical antidepressants, such as tricyclic antidepressants (TCAs), SSRIs, and serotonin‐noradrenaline reuptake inhibitors (SNRIs), mainly target monoamine neurotransmitter system.^87^ Nevertheless, 30% of the patients do not achieve remission with the available antidepressant drugs, and a big percentage of treated patients present adverse reactions. Now, some novel drugs, such as Baicalin, Memantine, XiaoYaoSan, Oridonin, Brexanolone, Zuranolone, Ketamine, etc., have also been used clinically on patients with depression.^88^, ^89^

Considering the important roles of mitochondrial disturbances in the development of depression, many researchers have been studying on the relationship between antidepressant mechanisms of the drugs and mitochondria. It has been demonstrated that some recent FDA‐approved antidepressants, such as Brexanolone and Zuranolone, can stimulate the production of endogenous neurosteroids through the translocator protein 18 kDa (TSPO), which is a transmembrane protein located primarily in mitochondria. And the neurosteroidogenic capacity of mitochondria and their regulation by TSPO may promise avenue for innovative therapeutic approaches within the complex interplay of factors contributing to depression.^90^ Moreover, Chen et al.^91^ demonstrated that Vortioxetine can rapidly form functional synapses in hippocampus of rats with mitochondrial support, which may be one of the reasons for its antidepressant effect. Additionally, it has been reported that Agomelatine can stabilize mitochondrial membrane stability through the Bax/Bcl2 balance and alleviate hippocampus damage in aging rats induced by D‐galactose.^92^ However, in fact, more studies on the effects of antidepressants on mitochondria are still focus on energy metabolism and quality control systems. (Table 2).

**TABLE 2:** The antidepressants related to mitochondria.

Name	Drug targets	Types of antidepressant	Effects on mitochondria
Brexanolone	GABAA receptors	GABAA receptor agonists	The production of endogenous neurosteroids through TSPO ↑
Zuranolone	GABAA receptors	GABAA receptor positive allosteric modulators	The production of endogenous neurosteroids through TSPO ↑
Vortioxetine	5‐HT receptors + SERT	5‐HT_1B_, 5‐HT_1A_ receptors agonists +5‐HT_3_, 5‐HT_7_, 5‐HT_1D_ receptor antagonists + SERT inhibitors	The number of mitochondria in the hippocampus ↑
Agomelatine	5‐HT_2C_ receptors + MTNR	5‐HT_2C_ receptor antagonists + MTNR agonists	Mitochondrial membrane stability ↑
Tianeptine	SERT	Atypical tricyclic antidepressants	The expression of mitochondrial energy metabolism‐related enzymes, such as Atp5b, Atp5o, and Cytc ↑
Paroxetine	SERT	Selective serotonin reuptake Inhibitors (SSRIs)	Mitochondrial respiratory chain complex I and complex II activity ↑
Venlafaxine	NET + SERT	SNRIs	Mitochondrial respiratory chain complex II and complex IV activity ↑
Baicalin	LSD1	Traditional Chinese medicines	Mitochondrial respiratory chain complex I and complex V activities ↑
Memantine	NMDA receptors	NMDA receptor antagonists	Mitochondrial respiratory chain complex I and complex V activities ↑
Fluoxetine	SERT	SSRIs	The expression of pyruvate dehydrogenase complex, mitochondrial DJ‐1, and NIX ↑
XiaoYaoSan	–	Traditional Chinese medicines	Lactate dehydrogenase activity ↑
Resveratrol	SIRT1	SIRT1 agonists	The expression of mitochondrial biogenesis related genes, such as SIRT1 and PGC‐1α ↑
Metformin	PRKAB1	AMPK activators	The expression of mitochondrial biogenesis related genes, such as NRF1, and TFAM ↑
Ketamine	NMDA receptors	NMDA receptor antagonists	The expression of mitophagy related genes, NIX ↑
Oridonin	–	Traditional Chinese medicines	Mitophagy ↑
Spilanthes acmella Murr	–	Traditional Chinese medicines	The expression of mitochondrial fission related genes, Drp1 ↓

Abbreviations: 5‐HT, 5‐hydroxytryptamine; AMPK, 5′‐AMP activated protein kinase; BNIP3L/NIX, BCL2 interacting protein 3 like; Drp1, Dynamin‐related protein 1; GABAA, gamma aminobutyric acid A; LSD1, lysine specific demethylase 1; MTNR, melatonin receptors; NET, norepinephrine transporter; NMDA, N‐methyl‐D‐aspartate; NRF1, nuclear respiratory factor 1; PGC‐1α, peroxisome proliferator‐activated receptor‐γ coactivator 1α; PRKAB1, 5′‐AMP‐activated protein kinase subunit beta‐1; SERT, serotonin transporter; SIRT1, silent information regulator‐1; SNRIs, serotonin‐noradrenaline reuptake inhibitors; SSRIs, selective serotonin reuptake inhibitors; TFAM, mitochondrial transcription factor A; TSPO, translocator protein 18 kDa.

### 5.1 Antidepressants improve the mitochondrial energy metabolism

As the abnormality of mitochondrial oxidative phosphorylation is closely associated with the pathogenesis of depression,^21^ multiple pieces of evidence suggest that antidepressants may ameliorate depression‐like behavior by enhancing the activity of the mitochondrial respiratory chain and the ATP production. For instance, it was observed that chronic Tianeptine administration up‐regulates the expression of mitochondrial energy metabolism‐related enzymes, such as Atp5b, Atp5o and Cytc, in hippocampal synaptosomal fractions of rats exposed to chronic social isolation.^93^ Moreover, Scaini et al.^37^ noted that the chronic administration of paroxetine increases the activities of mitochondrial respiratory chain complex I and complex II in the hippocampus, striatum, and cerebral cortex. They also reported that the administration of venlafaxine enhances mitochondrial respiratory chain complex II activity in the same brain regions, as well as increases complex IV activity only in the prefrontal cortex. Furthermore, it has been shown that novel drugs Baicalin and Memantine increase the activities of the mitochondrial respiratory chain complex I and complex V, as well as improve the mitochondrial function, and then enhance the ATP level in the brain of CUMS depressive mice.^30^, ^88^ These results support the conclusions that some antidepressants may exert their antidepressant effects at least partly by enhancing the expression of respiratory chain complexes and improving mitochondrial energy metabolism in brain. Inconsistent with the results, other studies reported that some antidepressants have different effects on mitochondria. Ľupták M et al.^94^ conducted a study demonstrating that the novel antipsychotics and antidepressants, including Brexpiprazole, Cariprazine, Loxapine, and Lurasidone, primarily inhibit individual ETC complexes and significantly decreased mitochondrial ATP production at higher concentrations. The paradox should be interpreted with caution due to the overdose of the drugs, which may lead to side effects of them. Notably, different correlation coefficients were observed for individual drugs concerning changes in the activity of specific ETC complexes and mitochondrial function, suggesting some specificity of the mitochondrial effects of individual psychopharmaca.^94^, ^95^ In addition, some studies in vivo have also shown that antidepressants often lead to upregulation of mitochondrial activity in various metabolic pathways during acute treatment, while chronic treatment leads to reduced or no change in mitochondrial activity.^94^, ^96^ Therefore, further work is needed to address this complexity.

It has been shown that some antidepressants can also affect other stages of aerobic glycolysis. For example, it has been demonstrated that chronic fluoxetine treatment enhances the expression of components of the pyruvate dehydrogenase complex in the frontal cortex of prenatal stress‐induced depressive rats.^97^ Wu et al.^98^ have also demonstrated that the antidepressant XiaoYaoSan increases the enzyme activity of lactate dehydrogenase in the hippocampus of CUMS rats, indicating that the antidepressant can modulate the disorders of glucose catabolism in chronic stress‐caused depressive rats. Of note, these preclinical studies linking mitochondrial energy metabolism and antidepression may provide a new insight to explore the new antidepressants and their underlying mechanisms.

### 5.2 Antidepressants amend the mitochondrial quality control systems

As impaired mitochondrial quality control systems are closely associated with depression,^66^ some studies revealed that some antidepressants can improve the abnormality of mitochondrial quality control systems in some kinds of animal models of depression. For instance, it has been demonstrated that chronic fluoxetine treatment upregulates the protein expression of the mitochondrial DJ‐1, which engaged in mitochondrial biogenesis, in the hippocampus of prenatally stressed offspring rats.^99^ Resveratrol, a naturally occurring polyphenolic antioxidant, is able to increase the mRNA expression of SIRT1 and PGC‐1α, and then promote mitochondrial biogenesis, eventually enhancing ATP level in the hippocampus of mice exposed to CUMS.^31^ A study by Lin et al.^100^ verified that Metformin significantly increases the mRNA levels of NRF1 and TFAM in the hippocampus of aged apoE4‐TR mice, performing the metformin‐mediated antidepressant‐like effect. These findings suggest it exists universally that antidepressant effects are consistent with improving mitochondrial biogenesis.

In mitophagy, previous studies have shown that fluoxetine increases the expression of NIX and enhances NIX‐mediated mitophagy in the hippocampus of CUMS mice.^65^, ^66^ Notably, Ketamine, a rapid on‐set and long‐lasting antidepressant, particularly in patients with treatment‐resistant depression, reverses the TNF‐α‐induced behavior despair through activation of NIX‐mediated mitophagy in the medial prefrontal cortex.^101^ Interestingly, Oridonin, the major active ingredient of the traditional Chinese medicinal herb Rabdosia rubescens, enhances mitophagy in LPS‐treated hippocampal astrocytes and attenuates cell death caused by ROS accumulation,^102^ which has been considered as one of the important causes of depression. Additionally, as for the mitochondrial fusion and fission, Suwanjang et al.^103^ demonstrated that Spilanthes acmella Murr. Extract significantly decreases the level of Drp1 in the hippocampus of chronic restraint stress rats. Based on the above information, it would imply that besides mitochondrial biogenesis, some antidepressants may also exert their antidepressant effects by enhancing mitophagy or balancing mitochondrial fusion and fission.

## 6 CONCLUSIONS AND FUTURE OUTLOOK

**FIGURE 2:**
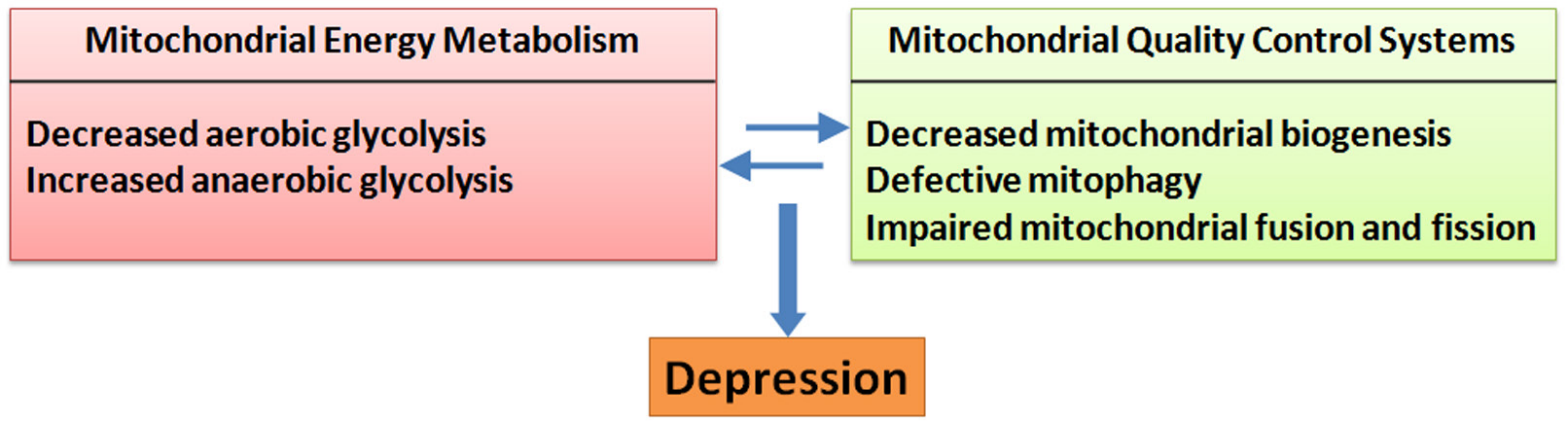
Simplified scheme of mitochondrial disorder as a mechanism underlying depression. Impaired brain energy metabolism and (or) damaged mitochondrial quality control systems may lead to depression.

This review emphasizes the vital roles of mitochondria in the pathogenesis of depression. It is noteworthy that impaired mitochondrial energy metabolism and damaged quality control systems have been proved to be involved in the development of depression (Figure 2), and some antidepressants exert their effects at least partly by improving mitochondrial function. This review may provide a new insight to study the underlying mechanisms in depression, and be of guiding value to explore new antidepressants specially designed to target mitochondria.

Despite these insights, there are still some gaps in our understanding of the mitochondrial mechanisms underlying depression. It is known that multiple mechanisms may contribute to depression, including monoamine neurotransmitter deficiency, hypothalamic–pituitary–adrenal (HPA) axis dysregulation, neuroinflammation over‐activation, etc., but how does the relationship between the mitochondrial mechanisms and other classical mechanisms of depression? Moreover, many factors have been found to be involved in the processes of mitochondrial energy metabolism and quality control systems, as well as play crucial roles in depression. What are the reasons for the varying expression of mitochondrial‐related proteins in different types of depression or depressive animal models? How to explain the association between mitochondrial dysfunction and the underlying signaling networks? The complex interplay between these pathways complicates the identification of specific targets for novel medication development. Difficult‐to‐treat depression, that is “treatment‐resistant depression”, is used for patients with depression symptoms that continue to cause significant burden despite the usual treatment efforts. It has been reported some patients with treatment‐resistant depression are diagnosed with mitochondrial encephalomyopathy with lactic acidosis and some novel antidepressants exert a rapid antidepressant effect in these patients in part by alleviating mitochondrial dysfunction.^104^, ^105^ Then, whether are the abnormalities of mitochondria and signaling networks similar in different types of depressive disorders, including treatment‐resistant depression? What are the differences among the effects of different antidepressants on mitochondria? Addressing these remaining questions would increase the understanding of mechanisms of depression and action of antidepressant drugs, and then aid the development of novel effective therapies to target specific mitochondrial functions.

## AUTHOR CONTRIBUTIONS

Writing—original draft preparation, Mengruo Jiang; writing—review and editing, Liyuan Wang and Hui Sheng; and supervision and funding acquisition, Hui Sheng. All authors have read and agreed to the published version of the manuscript.

## FUNDING INFORMATION

This work was supported by the Natural Science Foundation of China (No. 81671176).

## CONFLICT OF INTEREST STATEMENT

The authors declare no competing financial interests.

## Data Availability

Data sharing is not applicable to this article as no new data were created or analyzed in this study.

## References

[1] Kovich H , Kim W , Quaste AM . Pharmacologic treatment of depression. Am Fam Physician. 2023;107:173‐181. 36791444

[2] Chodavadia P , Teo I , Poremski D , Fung DSS , Finkelstein EA . Prevalence and economic burden of depression and anxiety symptoms among Singaporean adults: results from a 2022 web panel. BMC Psychiatry. 2023;23:104. 36782116 10.1186/s12888-023-04581-7 PMC9925363

[3] Li X , Huan J , Lin L , Hu Y . Association of systemic inflammatory biomarkers with depression risk: results from National Health and nutrition examination survey 2005–2018 analyses. Front Psych. 2023;14:1097196. 10.3389/fpsyt.2023.1097196 PMC9945233 36846218

[4] Büttiker P , Weissenberger S , Esch T , et al. Dysfunctional mitochondrial processes contribute to energy perturbations in the brain and neuropsychiatric symptoms. Front Pharmacol. 2022;13:1095923. 36686690 10.3389/fphar.2022.1095923 PMC9849387

[5] Ryan KM , Doody E , McLoughlin DM . Whole blood mitochondrial DNA copy number in depression and response to electroconvulsive therapy. Prog Neuropsychopharmacol Biol Psychiatry. 2023;121:110656. 36216200 10.1016/j.pnpbp.2022.110656

[6] Iwata R , Casimir P , Erkol E , et al. Mitochondria metabolism sets the species‐specific tempo of neuronal development. Science. 2023;379:eabn4705. 36705539 10.1126/science.abn4705

[7] Damri O , Natour S , Asslih S , Agam G . Does treatment with autophagy‐enhancers and/or ROS‐scavengers alleviate behavioral and neurochemical consequences of low‐dose rotenone‐induced mild mitochondrial dysfunction in mice? Mol Psychiatry. 2023;28:1667‐1678. 36690794 10.1038/s41380-023-01955-x PMC10208973

[8] Du F , Yu Q , Swerdlow RH , et al. Glucocorticoid‐driven mitochondrial damage stimulates tau pathology. Brain. 2023;146:4378‐4394. 37070763 10.1093/brain/awad127 PMC10545530

[9] Visentin APV , Colombo R , Scotton E , et al. Targeting inflammatory‐mitochondrial response in major depression: current evidence and further challenges. Oxid Med Cell Longev. 2020;2020:2972968. 32351669 10.1155/2020/2972968 PMC7178465

[10] Cataldo AM , McPhie DL , Lange NT , et al. Abnormalities in mitochondrial structure in cells from patients with bipolar disorder. Am J Pathol. 2010;177:575‐585. 20566748 10.2353/ajpath.2010.081068 PMC2913344

[11] Wu T , Huang Y , Gong Y , et al. Treadmill exercise ameliorates depression‐like behavior in the rats with prenatal dexamethasone exposure: the role of hippocampal mitochondria. Front Neurosci. 2019;13:264. 30971882 10.3389/fnins.2019.00264 PMC6443890

[12] Gebara E , Zanoletti O , Ghosal S , et al. Mitofusin‐2 in the nucleus Accumbens regulates anxiety and depression‐like behaviors through mitochondrial and neuronal actions. Biol Psychiatry. 2021;89:1033‐1044. 33583561 10.1016/j.biopsych.2020.12.003

[13] Khanra S , Reddy P , Giménez‐Palomo A , et al. Metabolic regulation to treat bipolar depression: mechanisms and targeting by trimetazidine. Mol Psychiatry. 2023;28:3231‐3242. 37386057 10.1038/s41380-023-02134-8 PMC10618096

[14] Li Y , Li J , Yang L , et al. Ginsenoside Rb1 protects hippocampal neurons in depressed rats based on mitophagy‐regulated astrocytic pyroptosis. Phytomedicine. 2023;121:155083. 37722244 10.1016/j.phymed.2023.155083

[15] Du Y , Gao F , Sun H , et al. Novel substituted 4‐(Arylethynyl)‐Pyrrolo[2,3‐d]pyrimidines negative allosteric modulators (NAMs) of the metabotropic glutamate receptor subtype 5 (mGlu5) treat depressive disorder in mice. Eur J Med Chem. 2023;261:115855. 37847955 10.1016/j.ejmech.2023.115855

[16] Liu R , Zhou H , Qu H , et al. Effects of aerobic exercise on depression‐like behavior and TLR4/NLRP3 pathway in hippocampus CA1 region of CUMS‐depressed mice. J Affect Disord. 2023;341:248‐255. 37634821 10.1016/j.jad.2023.08.078

[17] Shu X , Sun Y , Sun X , et al. The effect of fluoxetine on astrocyte autophagy flux and injured mitochondria clearance in a mouse model of depression. Cell Death Dis. 2019;10:577. 31371719 10.1038/s41419-019-1813-9 PMC6675792

[18] Ling‐Hu T , Liu SB , Gao Y , et al. Stable isotope‐resolved metabolomics reveals the abnormal brain glucose catabolism in depression based on chronic unpredictable mild stress rats. J Proteome Res. 2021;20:3549‐3558. 34077228 10.1021/acs.jproteome.1c00155

[19] Chen J , Xu W , Wang Z , et al. Mitochondrial pyruvate carrier influences ganoderic acid biosynthesis in *Ganoderma lucidum* . Appl Microbiol Biotechnol. 2023;107:1361‐1371. 36635397 10.1007/s00253-022-12357-4

[20] Wang B , Shi H , Yang B , et al. The mitochondrial Ahi1/GR participates the regulation on mtDNA copy numbers and brain ATP levels and modulates depressive behaviors in mice. Cell Commun Signal. 2023;21:21. 36691038 10.1186/s12964-022-01034-8 PMC9869592

[21] Głombik K , Kukla‐Bartoszek M , Curzytek K , Detka J , Basta‐Kaim A , Budziszewska B . The effects of prenatal dexamethasone exposure on brain metabolic homeostasis in adulthood: implications for depression. Int J Mol Sci. 2023;24:1156. 36674678 10.3390/ijms24021156 PMC9866429

[22] Su L , Cai Y , Xu Y , Dutt A , Shi S , Bramon E . Cerebral metabolism in major depressive disorder: a voxel‐based meta‐analysis of positron emission tomography studies. BMC Psychiatry. 2014;14:321. 25407081 10.1186/s12888-014-0321-9 PMC4240898

[23] Moore CM , Christensen JD , Lafer B , Fava M , Renshaw PF . Lower levels of nucleoside triphosphate in the basal ganglia of depressed subjects: a phosphorous‐31 magnetic resonance spectroscopy study. Am J Psychiatry. 1997;154:116‐118. 8988971 10.1176/ajp.154.1.116

[24] Kious BM , Kondo DG , Renshaw PF . Creatine for the treatment of depression. Biomolecules. 2019;9:9. 10.3390/biom9090406 PMC6769464 31450809

[25] Delanogare E , Bullich S , Barbosa L , et al. Metformin improves neurobehavioral impairments of streptozotocin‐treated and western diet‐fed mice: beyond glucose‐lowering effects. Fundam Clin Pharmacol. 2023;37:94‐106. 35996325 10.1111/fcp.12825

[26] Ruegsegger GN , Vanderboom PM , Dasari S , et al. Exercise and metformin counteract altered mitochondrial function in the insulin‐resistant brain. JCI Insight. 2019;4:4. 10.1172/jci.insight.130681 PMC6795285 31534057

[27] Liu W , Sheng H , Xu Y , Liu Y , Lu J , Ni X . Swimming exercise ameliorates depression‐like behavior in chronically stressed rats: relevant to proinflammatory cytokines and IDO activation. Behav Brain Res. 2013;242:110‐116. 23291157 10.1016/j.bbr.2012.12.041

[28] Peng Z , Zhang C , Yan L , et al. EPA is more effective than DHA to improve depression‐like behavior, glia cell dysfunction and Hippcampal apoptosis signaling in a chronic stress‐induced rat model of depression. Int J Mol Sci. 2020;21:1769. 32150824 10.3390/ijms21051769 PMC7084382

[29] Li D , Liao Q , Tao Y , et al. Downregulation of CRTC1 is involved in CUMS‐induced depression‐like behavior in the hippocampus and its RNA sequencing analysis. Mol Neurobiol. 2022;59:4405‐4418. 35556215 10.1007/s12035-022-02787-6

[30] Lu S , Li C , Jin X , et al. Baicalin improves the energy levels in the prefrontal cortex of mice exposed to chronic unpredictable mild stress. Heliyon. 2022;8:e12083. 36531636 10.1016/j.heliyon.2022.e12083 PMC9747579

[31] Shen JD , Zhang YW , Wang BY , et al. Effects of resveratrol on the levels of ATP, 5‐HT and GAP‐43 in the hippocampus of mice exposed to chronic unpredictable mild stress. Neurosci Lett. 2020;735:135232. 32621948 10.1016/j.neulet.2020.135232

[32] Cao X , Li LP , Wang Q , et al. Astrocyte‐derived ATP modulates depressive‐like behaviors. Nat Med. 2013;19:773‐777. 23644515 10.1038/nm.3162

[33] Alhassen S , Chen S , Alhassen L , et al. Intergenerational trauma transmission is associated with brain metabotranscriptome remodeling and mitochondrial dysfunction. Commun Biol. 2021;4:783. 34168265 10.1038/s42003-021-02255-2 PMC8225861

[34] Laugesen K , Sørensen HT , Jørgensen JOL , Petersen I . In utero exposure to glucocorticoids and risk of anxiety and depression in childhood or adolescence. Psychoneuroendocrinology. 2022;141:105766. 35447494 10.1016/j.psyneuen.2022.105766

[35] Rezin GT , Amboni G , Zugno AI , Quevedo J , Streck EL . Mitochondrial dysfunction and psychiatric disorders. Neurochem Res. 2009;34:1021‐1029. 18979198 10.1007/s11064-008-9865-8

[36] Holper L , Ben‐Shachar D , Mann JJ . Multivariate meta‐analyses of mitochondrial complex I and IV in major depressive disorder, bipolar disorder, schizophrenia, Alzheimer disease, and Parkinson disease. Neuropsychopharmacology. 2019;44:837‐849. 29855563 10.1038/s41386-018-0090-0 PMC6461987

[37] Scaini G , Maggi DD , De‐Nês BT , et al. Activity of mitochondrial respiratory chain is increased by chronic administration of antidepressants. Acta Neuropsychiatr. 2011;23:112‐118. 26952897 10.1111/j.1601-5215.2011.00548.x

[38] Damri O , Asslih S , Shemesh N , et al. Using mitochondrial respiration inhibitors to design a novel model of bipolar disorder‐like phenotype with construct, face and predictive validity. Transl Psychiatry. 2021;11:123. 33579900 10.1038/s41398-021-01215-y PMC7881114

[39] Ben‐Shachar D , Karry R . Neuroanatomical pattern of mitochondrial complex I pathology varies between schizophrenia, bipolar disorder and major depression. PloS One. 2008;3:e3676. 18989376 10.1371/journal.pone.0003676 PMC2579333

[40] Khan M , Baussan Y , Hebert‐Chatelain E . Connecting dots between mitochondrial dysfunction and depression. Biomolecules. 2023;13:13. 10.3390/biom13040695 PMC10135685 37189442

[41] Goetzl EJ , Wolkowitz OM , Srihari VH , et al. Abnormal levels of mitochondrial proteins in plasma neuronal extracellular vesicles in major depressive disorder. Mol Psychiatry. 2021;26:7355‐7362. 34471251 10.1038/s41380-021-01268-x PMC8872999

[42] Bajek‐Bil A , Chmiel M , Włoch A , Stompor‐Gorący M . Baicalin‐current trends in detection methods and health‐promoting properties. Pharmaceuticals (Basel). 2023;16:570. 37111327 10.3390/ph16040570 PMC10146343

[43] Linghu T , Zhao Y , Wu W , Gao Y , Tian J , Qin X . Novel targets for ameliorating energy metabolism disorders in depression through stable isotope‐resolved metabolomics. Biochim Biophys Acta Bioenerg. 2022;1863:148578. 35640666 10.1016/j.bbabio.2022.148578

[44] Filipović D , Perić I , Costina V , Stanisavljević A , Gass P , Findeisen P . Social isolation stress‐resilient rats reveal energy shift from glycolysis to oxidative phosphorylation in hippocampal nonsynaptic mitochondria. Life Sci. 2020;254:117790. 32416165 10.1016/j.lfs.2020.117790

[45] Wang L , Feng X , Ye C , Wang C , Wang M . Shen Shuai II recipe inhibits hypoxia‐induced glycolysis by preserving mitochondrial dynamics to attenuate kidney fibrosis. J Ethnopharmacol. 2023;308:116271. 36806483 10.1016/j.jep.2023.116271

[46] Belal S , Goudenège D , Bocca C , et al. Glutamate‐induced deregulation of Krebs cycle in mitochondrial encephalopathy lactic acidosis syndrome stroke‐like episodes (MELAS) syndrome is alleviated by ketone body exposure. Biomedicine. 2022;10:10. 10.3390/biomedicines10071665 PMC9312837 35884972

[47] Ernst J , Hock A , Henning A , Seifritz E , Boeker H , Grimm S . Increased pregenual anterior cingulate glucose and lactate concentrations in major depressive disorder. Mol Psychiatry. 2017;22:113‐119. 27184123 10.1038/mp.2016.73

[48] Bradley KA , Mao X , Case JA , et al. Increased ventricular cerebrospinal fluid lactate in depressed adolescents. Eur Psychiatry. 2016;32:1‐8. 26802978 10.1016/j.eurpsy.2015.08.009 PMC4831134

[49] Regenold WT , Phatak P , Marano CM , Sassan A , Conley RR , Kling MA . Elevated cerebrospinal fluid lactate concentrations in patients with bipolar disorder and schizophrenia: implications for the mitochondrial dysfunction hypothesis. Biol Psychiatry. 2009;65:489‐494. 19103439 10.1016/j.biopsych.2008.11.010 PMC3752997

[50] Liu X , Wei F , Liu H , Zhao S , du G , Qin X . Integrating hippocampal metabolomics and network pharmacology deciphers the antidepressant mechanisms of Xiaoyaosan. J Ethnopharmacol. 2021;268:113549. 33152435 10.1016/j.jep.2020.113549

[51] Detka J , Kurek A , Kucharczyk M , et al. Brain glucose metabolism in an animal model of depression. Neuroscience. 2015;295:198‐208. 25819664 10.1016/j.neuroscience.2015.03.046

[52] Surma M , Anbarasu K , Dutta S , et al. Enhanced mitochondrial biogenesis promotes neuroprotection in human pluripotent stem cell derived retinal ganglion cells. Commun Biol. 2023;6:218. 36828933 10.1038/s42003-023-04576-w PMC9957998

[53] Wu Y , Sun F , Guo Y , et al. Curcumin relieves chronic unpredictable mild stress‐induced depression‐like behavior through the PGC‐1α/FNDC5/BDNF pathway. Behav Neurol. 2021;2021:2630445. 34950248 10.1155/2021/2630445 PMC8692045

[54] Scaini G , Mason BL , Diaz AP , et al. Dysregulation of mitochondrial dynamics, mitophagy and apoptosis in major depressive disorder: does inflammation play a role? Mol Psychiatry. 2022;27:1095‐1102. 34650203 10.1038/s41380-021-01312-w

[55] Yan L , Liu CH , Xu L , et al. Alpha‐Asarone modulates kynurenine disposal in muscle and mediates resilience to stress‐induced depression via PGC‐1α induction. CNS Neurosci Ther. 2023;29:941‐956. 36575869 10.1111/cns.14030 PMC9928554

[56] Ryan KM , Patterson I , McLoughlin DM . Peroxisome proliferator‐activated receptor gamma co‐activator‐1 alpha in depression and the response to electroconvulsive therapy. Psychol Med. 2019;49:1859‐1868. 30191781 10.1017/S0033291718002556

[57] Alcocer‐Gómez E , Núñez‐Vasco J , Casas‐Barquero N , et al. Gene expression profile in major depressive disorder shows reduced mitochondrial biogenesis. CNS Neurosci Ther. 2016;22:636‐638. 27234291 10.1111/cns.12568 PMC6492842

[58] Hu S , He L , Chen B , You Y . Apelin‐13 attenuates depressive‐like behaviors induced by chronic unpredictable mild stress via activating AMPK/PGC‐1α/FNDC5/BDNF pathway. Peptides. 2022;156:170847. 35908670 10.1016/j.peptides.2022.170847

[59] Yang C , Sui G , Li D , et al. Exogenous IGF‐1 alleviates depression‐like behavior and hippocampal mitochondrial dysfunction in high‐fat diet mice. Physiol Behav. 2021;229:113236. 33137345 10.1016/j.physbeh.2020.113236

[60] Alhaddad A , Radwan A , Mohamed NA , et al. Rosiglitazone mitigates dexamethasone‐induced depression in mice via modulating brain glucose metabolism and AMPK/mTOR signaling pathway. Biomedicine. 2023;11:11. 10.3390/biomedicines11030860 PMC10046017 36979839

[61] Lei Y , Wang J , Wang D , et al. SIRT1 in forebrain excitatory neurons produces sexually dimorphic effects on depression‐related behaviors and modulates neuronal excitability and synaptic transmission in the medial prefrontal cortex. Mol Psychiatry. 2020;25:1094‐1111. 30705425 10.1038/s41380-019-0352-1 PMC7192847

[62] Song J , Shen B , Yang YJ , et al. Non‐motor symptoms in Parkinson's disease patients with parkin mutations: more depression and less executive dysfunction. J Mol Neurosci. 2020;70:246‐253. 31927768 10.1007/s12031-019-01444-3

[63] Agnihotri SK , Sun L , Yee BK , et al. PINK1 deficiency is associated with increased deficits of adult hippocampal neurogenesis and lowers the threshold for stress‐induced depression in mice. Behav Brain Res. 2019;363:161‐172. 30735759 10.1016/j.bbr.2019.02.006

[64] Rodkin S , Nwosu C , Sannikov A , et al. The role of Gasotransmitter‐dependent signaling mechanisms in apoptotic cell death in cardiovascular, rheumatic, kidney, and neurodegenerative diseases and mental disorders. Int J Mol Sci. 2023;24:6014. 37046987 10.3390/ijms24076014 PMC10094524

[65] Choi GE , Lee HJ , Chae CW , et al. BNIP3L/NIX‐mediated mitophagy protects against glucocorticoid‐induced synapse defects. Nat Commun. 2021;12:487. 33473105 10.1038/s41467-020-20679-y PMC7817668

[66] Jin X , Zhu L , Lu S , et al. Baicalin ameliorates CUMS‐induced depression‐like behaviors through activating AMPK/PGC‐1α pathway and enhancing NIX‐mediated mitophagy in mice. Eur J Pharmacol. 2023;938:175435. 36463946 10.1016/j.ejphar.2022.175435

[67] Feng Z , Zou X , Jia H , et al. Maternal docosahexaenoic acid feeding protects against impairment of learning and memory and oxidative stress in prenatally stressed rats: possible role of neuronal mitochondria metabolism. Antioxid Redox Signal. 2012;16:275‐289. 21905985 10.1089/ars.2010.3750

[68] Liu W , Zhou C . Corticosterone reduces brain mitochondrial function and expression of mitofusin, BDNF in depression‐like rodents regardless of exercise preconditioning. Psychoneuroendocrinology. 2012;37:1057‐1070. 22244747 10.1016/j.psyneuen.2011.12.003

[69] Chen C , Wang Y , Zhang J , Ma L , Gu J , Ho G . Contribution of neural cell death to depressive phenotypes of streptozotocin‐induced diabetic mice. Dis Model Mech. 2014;7:723‐730. 24764190 10.1242/dmm.016162 PMC4036479

[70] Tabassum S , Misrani A , Huo Q , Ahmed A , Long C , Yang L . Minocycline ameliorates chronic unpredictable mild stress‐induced neuroinflammation and abnormal mPFC‐HIPP oscillations in mice. Mol Neurobiol. 2022;59:6874‐6895. 36048340 10.1007/s12035-022-03018-8

[71] Deng D , Cui Y , Gan S , et al. Sinisan alleviates depression‐like behaviors by regulating mitochondrial function and synaptic plasticity in maternal separation rats. Phytomedicine. 2022;106:154395. 36103769 10.1016/j.phymed.2022.154395

[72] Figueiredo‐Pereira C , Villarejo‐Zori B , Cipriano PC , et al. Carbon monoxide stimulates both mitophagy and mitochondrial biogenesis to mediate protection against oxidative stress in astrocytes. Mol Neurobiol. 2023;60:851‐863. 36378469 10.1007/s12035-022-03108-7

[73] Głombik K , Stachowicz A , Ślusarczyk J , et al. Maternal stress predicts altered biogenesis and the profile of mitochondrial proteins in the frontal cortex and hippocampus of adult offspring rats. Psychoneuroendocrinology. 2015;60:151‐162. 26143539 10.1016/j.psyneuen.2015.06.015

[74] Hu N , Chen X , Chen C , et al. Exploring the role of esketamine in alleviating depressive symptoms in mice via the PGC‐1α/irisin/ERK1/2 signaling pathway. Sci Rep. 2023;13:16611. 37789092 10.1038/s41598-023-43684-9 PMC10547795

[75] Kim HD , Hesterman J , Call T , et al. SIRT1 mediates depression‐like behaviors in the nucleus Accumbens. J Neurosci. 2016;36:8441‐8452. 27511015 10.1523/JNEUROSCI.0212-16.2016 PMC4978803

[76] Zhang Y , Fang Q , Wang H , et al. Increased mitophagy protects cochlear hair cells from aminoglycoside‐induced damage. Autophagy. 2023;19:75‐91. 35471096 10.1080/15548627.2022.2062872 PMC9809934

[77] Liu Y , Wang M , Hou XO , Hu LF . Roles of microglial mitophagy in neurological disorders. Front Aging Neurosci. 2022;14:979869. 36034136 10.3389/fnagi.2022.979869 PMC9399802

[78] Wang G , Liu Y , Zhu X , et al. Knockdown of miRNA‐134‐5p rescues dendritic deficits by promoting AMPK‐mediated mitophagy in a mouse model of depression. Neuropharmacology. 2022;214:109154. 35659969 10.1016/j.neuropharm.2022.109154

[79] Li J , Yang D , Li Z , et al. PINK1/parkin‐mediated mitophagy in neurodegenerative diseases. Ageing Res Rev. 2023;84:101817. 36503124 10.1016/j.arr.2022.101817

[80] Cao Y , Zheng J , Wan H , et al. A mitochondrial SCF‐FBXL4 ubiquitin E3 ligase complex degrades BNIP3 and NIX to restrain mitophagy and prevent mitochondrial disease. EMBO J. 2023;42:e113033. 36896912 10.15252/embj.2022113033 PMC10308365

[81] Zargani M , Rahimi A , Mazaheri Tirani Z , Arabzadeh E , Feizolahi F . Swimming exercise and nano‐l‐arginine supplementation improve oxidative capacity and some autophagy‐related genes in the soleus muscle of aging rats. Gene. 2023;850:146955. 36220447 10.1016/j.gene.2022.146955

[82] Schuettpelz J , Janer A , Antonicka H , et al. The role of the mitochondrial outer membrane protein SLC25A46 in mitochondrial fission and fusion. Life Sci Alliance. 2023;6:e202301914. 36977595 10.26508/lsa.202301914 PMC10052876

[83] Hu SL , Mamun AA , Shaw J , et al. TBK1‐medicated DRP1 phosphorylation orchestrates mitochondrial dynamics and autophagy activation in osteoarthritis. Acta Pharmacol Sin. 2023;44:610‐621. 36008706 10.1038/s41401-022-00967-7 PMC9958127

[84] Brivio P , Audano M , Gallo MT , et al. Metabolomic signature and mitochondrial dynamics outline the difference between vulnerability and resilience to chronic stress. Transl Psychiatry. 2022;12:87. 35228511 10.1038/s41398-022-01856-7 PMC8885712

[85] Vevea JD , Chapman ER . Mitofusin 2 sustains the axonal mitochondrial network to support presynaptic Ca(2+) homeostasis and the synaptic vesicle cycle in rat hippocampal axons. J Neurosci. 2023;43:3421‐3438. 36997314 10.1523/JNEUROSCI.1356-22.2023 PMC10175236

[86] Wang J , Yang Y , Sun F , et al. ALKBH5 attenuates mitochondrial fission and ameliorates liver fibrosis by reducing Drp1 methylation. Pharmacol Res. 2023;187:106608. 36566000 10.1016/j.phrs.2022.106608

[87] Malhi GS , Acar M , Kouhkamari MH , et al. Antidepressant prescribing patterns in Australia. BJPsych Open. 2022;8:e120. 35770420 10.1192/bjo.2022.522 PMC9301763

[88] Mishra SK , Hidau MK , Rai S . Memantine treatment exerts an antidepressant‐like effect by preventing hippocampal mitochondrial dysfunction and memory impairment via upregulation of CREB/BDNF signaling in the rat model of chronic unpredictable stress‐induced depression. Neurochem Int. 2021;142:104932. 33290797 10.1016/j.neuint.2020.104932

[89] Wang XL , Feng ST , Wang YT , Zhang NN , Wang ZZ , Zhang Y . Canonical Chinese medicine formula Danzhi‐Xiaoyao‐San for treating depression: a systematic review and meta‐analysis. J Ethnopharmacol. 2022;287:114960. 34968660 10.1016/j.jep.2021.114960

[90] Rupprecht R , Wetzel CH , Dorostkar M , et al. Translocator protein (18 kDa) TSPO: a new diagnostic or therapeutic target for stress‐related disorders? Mol Psychiatry. 2022;27:2918‐2926. 35444254 10.1038/s41380-022-01561-3

[91] Chen F , Danladi J , Ardalan M , et al. A critical role of mitochondria in BDNF‐associated synaptic plasticity after one‐week vortioxetine treatment. Int J Neuropsychopharmacol. 2018;21:603‐615. 29514282 10.1093/ijnp/pyy022 PMC6007239

[92] Chanmanee T , Wongpun J , Tocharus C , Govitrapong P , Tocharus J . The effects of agomelatine on endoplasmic reticulum stress related to mitochondrial dysfunction in hippocampus of aging rat model. Chem Biol Interact. 2022;351:109703. 34673010 10.1016/j.cbi.2021.109703

[93] Perić I , Costina V , Djordjević S , et al. Tianeptine modulates synaptic vesicle dynamics and favors synaptic mitochondria processes in socially isolated rats. Sci Rep. 2021;11:17747. 34493757 10.1038/s41598-021-97186-7 PMC8423821

[94] Ľupták M , Fišar Z , Hroudová J . Effect of novel antipsychotics on energy metabolism ‐ in vitro study in pig brain mitochondria. Mol Neurobiol. 2021;58:5548‐5563. 34365585 10.1007/s12035-021-02498-4

[95] Fizíková I , Dragašek J , Račay P . Mitochondrial dysfunction, altered mitochondrial oxygen, and energy metabolism associated with the pathogenesis of schizophrenia. Int J Mol Sci. 2023;24:7991. 37175697 10.3390/ijms24097991 PMC10178941

[96] Chan ST , McCarthy MJ , Vawter MP . Psychiatric drugs impact mitochondrial function in brain and other tissues. Schizophr Res. 2020;217:136‐147. 31744750 10.1016/j.schres.2019.09.007 PMC7228833

[97] Głombik K , Stachowicz A , Trojan E , et al. Mitochondrial proteomics investigation of frontal cortex in an animal model of depression: focus on chronic antidepressant drugs treatment. Pharmacol Rep. 2018;70:322‐330. 29477041 10.1016/j.pharep.2017.11.016

[98] Wu WZ , Ling‐Hu T , Zhao YH , et al. A unique insight for Xiaoyao San exerts antidepressant effects by modulating hippocampal glucose catabolism using stable isotope‐resolved metabolomics. J Ethnopharmacol. 2023;300:115702. 36099982 10.1016/j.jep.2022.115702

[99] Głombik K , Stachowicz A , Trojan E , et al. Evaluation of the effectiveness of chronic antidepressant drug treatments in the hippocampal mitochondria – a proteomic study in an animal model of depression. Prog Neuropsychopharmacol Biol Psychiatry. 2017;78:51‐60. 28526399 10.1016/j.pnpbp.2017.05.014

[100] Lin Y , Dai X , Zhang J , Chen X . Metformin alleviates the depression‐like behaviors of elderly apoE4 mice via improving glucose metabolism and mitochondrial biogenesis. Behav Brain Res. 2022;423:113772. 35090900 10.1016/j.bbr.2022.113772

[101] Lu JJ , Wu PF , He JG , et al. BNIP3L/NIX‐mediated mitophagy alleviates passive stress‐coping behaviors induced by tumor necrosis factor‐α. Mol Psychiatry. 2023. Online ahead of print. 10.1038/s41380-023-02008-z 36914810

[102] Li C , Zhu Y , Wu Y , et al. Oridonin alleviates LPS‐induced depression by inhibiting NLRP3 inflammasome via activation of autophagy. Front Med (Lausanne). 2021;8:813047. 35096901 10.3389/fmed.2021.813047 PMC8790066

[103] Suwanjang W , Ruankham W , Chetsawang B , et al. Spilanthes acmella Murr. Ameliorates chronic stress through improving mitochondrial function in chronic restraint stress rats. Neurochem Int. 2021;148:105083. 34052298 10.1016/j.neuint.2021.105083

[104] Anglin RE , Garside SL , Tarnopolsky MA , Mazurek MF , Rosebush PI . The psychiatric manifestations of mitochondrial disorders: a case and review of the literature. J Clin Psychiatry. 2012;73:506‐512. 22579150 10.4088/JCP.11r07237

[105] Anglin RE , Tarnopolsky MA , Mazurek MF , Rosebush PI . The psychiatric presentation of mitochondrial disorders in adults. J Neuropsychiatry Clin Neurosci. 2012;24:394‐409. 23224446 10.1176/appi.neuropsych.11110345

